# Antibody-Drug Conjugates: Possibilities and Challenges

**Published:** 2019

**Authors:** Mohammad-Reza Nejadmoghaddam, Arash Minai-Tehrani, Ramin Ghahremanzadeh, Morteza Mahmoudi, Rassoul Dinarvand, Amir-Hassan Zarnani

**Affiliations:** 1. Nanotechnology Research Center, Faculty of Pharmacy, Tehran University of Medical Sciences, Tehran, Iran; 2. Nanobiotechnology Research Center, Avicenna Research Institute, ACECR, Tehran, Iran; 3. Department of Pharmaceutics, Faculty of Pharmacy, Tehran University of Medical Sciences, Tehran, Iran; 4. Department of Immunology, Faculty of Public Health, Tehran University of Medical Sciences, Tehran, Iran; 5. Reproductive Immunology Research Center, Avicenna Research Institute, ACECR, Tehran, Iran; 6. Immunology Research Center, Iran University of Medical Sciences, IUMS, Tehran, Iran

**Keywords:** Antibody-Drug, Cancer therapy, Cytotoxic drugs, Monoclonal antibodies, Nanomedicine

## Abstract

The design of Antibody Drug Conjugates (ADCs) as efficient targeting agents for tumor cell is still in its infancy for clinical applications. This approach incorporates the antibody specificity and cell killing activity of chemically conjugated cytotoxic agents. Antibody in ADC structure acts as a targeting agent and a nanoscale carrier to deliver a therapeutic dose of cytotoxic cargo into desired tumor cells. Early ADCs encountered major obstacles including, low blood residency time, low penetration capacity to tumor microenvironment, low payload potency, immunogenicity, unusual off-target toxicity, drug resistance, and the lack of stable linkage in blood circulation. Although extensive studies have been conducted to overcome these issues, the ADCs based therapies are still far from having high-efficient clinical outcomes. This review outlines the key characteristics of ADCs including tumor marker, antibody, cytotoxic payload, and linkage strategy with a focus on technical improvement and some future trends in the pipeline.

## Introduction

**Figure 1.**
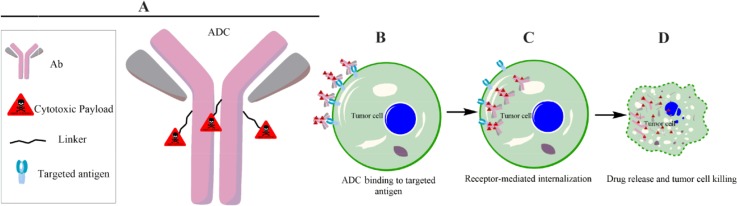
Schematic representation of ADC, showing the main components of an ADC and its cell cytotoxicity mechanism. Clinical efficacy of ADCs is determined by fine-tuning combination of tumor antigen, targeting antibody, cytotoxic payload and conjugation strategy (a). ADC binds to tumor target cell surface antigens (b) leading to trigger a specific receptor mediated internalization (c). The internalized ADCs are decomposed to release cytotoxic payloads inside the tumor cell either through its linkage/linker sensitivity to protease, acidic, reductive agents or by lysosomal process, leading to cell death (d).

Similar to conventional cancer treatments such as chemotherapy and radiotherapy, antibody immunotherapy and targeted therapies based on nanoparticulate structures are not safe and efficacious as often claimed; therefore, alternative therapies are urgently needed. In this regard, Antibody Drug Conjugates (ADC) technology that could bring forth a new generation of cancer therapeutics was the main focus of this study. ADCs are monoclonal antibodies (mAbs) connected by a specified linkage to antitumor cytotoxic molecules. The main components of an ADC and mechanism of its action are further demonstrated in figure 1.

In ADC technology, the specificity of an antibody for its immunogenicity is exploited to home a chemically supertoxic agent into tumor cells, while administration of unconjugated drug alone is not suitable due to its high toxicity. Therefore, ADCs can be further defined as prodrugs requiring the release of their toxic agent for their activation that commonly happens after ADC internalization into the target cell 1. From the standpoint of nanomedicine, the antibody in ADC structure acts as a self-targeting nanoscale carrier 1–3, thus, it could overcome the issues associated with nanomedicines based on synthetic nanomaterials such as cellular internalization, clearance, sterical hindering of binding to the epitopes and failing to release into targeted cells 4.

The first experimental design on ADC subject dates back to more than 50 years ago 5. However, the use of ADCs for cancer therapy has achieved considerable success in recent years after the introduction of four clinically approved ADCs such as Brentuximab vedotin 6,7, Trastuzumab emtansine 8–11, Inotuzumab ozogamicin 12 and Gemtuzumab ozogamicin 12,13 used for the treatment of patients with lymphoma (HL and ALL), HER2-positive, CD22-positive AML and CD33-positive ALL cancers, respectively. Likewise, a great deal of effort has also been made by the pharmaceutical companies to overcome the technological barriers associated with ADCs 14,15, whereby there are 160 ADCs undergoing preclinical trials 16 and 70 more under various stages of clinical evaluation (Table 1).

**Table 1.** Current ADCs in clinical development based on targeting antigens with an overview of their properties

**ADC names**	**Clinical phase, indication**	**Ab, kd, therapeutics activity**	**Payload**	**Linkage strategy**	**DAR, MTD, bystander effect**	**Sponsor, Reference**
**Targeting HER2 antigen, a transmembrane RTKs in the growth of some cancer cells:**						
Kadcyla, Ado-Trastuzumab emtansine, T-DM1	Approved in 2013, for treatment of her2 positive breast cancer	huIgG1 (trastuzumab), n/a, ADCC and HER2-dependent PI3K/AKT signaling	DM1	Native lysine residues, SMCC nonreducible thioether linkage	∼3.5, 3.6 *mg/kg*, no	Genentech, Inc. (8–11)
SYD985, Trastuzumab vc-seco-DUBA	Phase I, for treatment of USC and epithelial EOC	huIgG2 anti HER2 (Trastuzumab), n/a, no	DUO	VC-seco	∼ 2.8, 1.88 *mg/kg*, yes	Synthon BV (37–39)
ADC XMT-1522	Phase I, for treatment of low HER+ breast, gastric and lung cancers	huIgG1anti-HER2 (HT-19), n/a, n/a	AF-HPA	Fleximer®	12, n/a, yes	Mersana Therapeutics (40)
ADC ARX788	Phase I, for treatment of low HER+ breast, ovarian, lung and gastric cancers	IgG1anti-HER2, n/a, n/a	MMAF	pAcF site-specific oxime linkage, AS269 noncleavable linker	2, n/a, n/a	Zhejiang Medicine/Ambrx (41)
ADC ADCT-502	Phase I, for treatment of low HER2+ expressing breast, NSCLC, gastroesophageal, bladder cancer	huIgG1anti-HER2 (trastuzumab)	PBD	Cysteine residues, VA-PABC	1.7, n/a, n/a	ADC Therapeutics S.A. (42)
**Targeting EGFR antigen, a RTKs that is essential for ductal and lobuloalveolar development:**						
ABT-414, Depatuxizumab mafodotin	Phase II, for treatment of GBM	huIgG1 anti EGFR (ABT-806), 0.06 *nM*, inhibits EGFR signaling	MMAF	Native cysteine residues, MC noncleavable linker	∼3.8,1.5 *mg/kg*, no	Abbvie (43)
AMG 595	Phase I, for treatment of GBM	huIgG1anti-EGFRvIII, 0.61 *nM*, n/a	DM1	Native lysine residues, SMCC noncleavable thioether linker	∼3.5, n/a, no	Amgen (44)
IMGN289, Laprituximab emtansine	Phase I, for treatment of NSCLC and HNSCC	huIgG anti-EGFR (J2898A), n/a, n/a	DM1	Native lysine residues, SMCC noncleavable thioether linker	n/a, n/a, no	ImmunoGen (45)
ABBV-221	Phase I, for treatment of solid tumor	huIgG1 anti-EGFR, n/a, n/a	MMAE	VC protease-cleavable linker	n/a, n/a, n/a	Abbvie (46)
**Targeting CD70 (CD27L) antigen a TP2 and member of the tumor necrosis factor family:**						
SGN-75	Phase I, for treatment of CD70-positive NHL and metastatic RCC	hu anti-CD70 (h1F6), n/a, n/a	MMAF	Native cysteine residues, MC noncleavable linker	n/a, 3, n/a	Seattle Genetics (47)
MDX-1203, BMS-936561	Phase I, for treatment of ccRCC or B-NHL	hu anti-CD70, n/a, n/a	DUO	Native cysteine residues, VC protease-cleavable linker	n/a,15 *mg/kg*, yes	Bristol-Myers (48)
SGN-CD70A	Phase I, for treatment of RCC, MCLD, LBC, FL,	hu anti-CD70, n/a, n/a	PBD	VA linker	n/a, n/a, yes	Seattle Genetics (49)
AMG 172	Phase I, for treatment of ccRCC	huIgG1, n/a, n/a	DM1	Native lysine residues, MCC noncleavable linker	n/a, n/a, no	Amgen (50)
**Targeting CD33 antigen, a EGP:**						
Mylotarg, Gemtuzumab Ozogamicin (GO)	Withdrawn 2010 and approved in 2017, for treatment of CD33^+^ AML	huIgG4, n/a, n/a	Calich.	Native lysine residues, (AcBut)-N-acyl acid-labile hydrazone linker	n/a, 0.25 *mg/kg*, yes	Pifizer (51)
SGN-CD33A	Phase I, for treatment of AML	hu anti-CD33 with engineered cysteines, n/a, n/a	PBD	Engineered cysteine residues, VA linker	n/a, n/a, yes	Seattle Genetics (12,13)
AVE9633	Phase I, for treatment of AML	anti-CD33, n/a, n/a	DM4	Native lysine residues, SPDB disulfide cleavable linker	n/a, n/a, n/a	Sanofi (53)
**Targeting CD19 antigen, a TP1 on B cells as an accessory molecule for B-cell signal transduction and TAA:**						
SAR3419, coltuximab ravtansine	Phase II, for treatment of B-NHL and B-ALL	huIgG1 anti-CD19 (huB4), n/a, ADCC	DM4	Native lysine residues, SPDB disulfide cleavable linker	∼3.5, ∼4.3 *mg/kg*, yes	ImmunoGen (7,34,35)
SGN-CD19A	Phase I, for treatment of B-Cell Malignancies	huIgG1 anti-CD19 (hBU12), n/a, ADCC	MMAF	Native cysteine residues, MC linker, noncleavable	n/a, 6.0, no	Seattle Genetics (32)
ADCT-402	Phase I, for treatment of relapsed or refractory B-ALL	huIgG1anti-CD19, n/a, n/a	PBD	Native cysteine residues, VA and maleimide cleavable linker	n/a, n/a, n/a	ADC Therapeutics S.A. (33)
**Targeting Mesothelin antigen, a glycophosphatidyl inositol anchored protein:**						
BAY 94–9343, anetumab ravtansine	Phase II, for treatment of MPM	hu anti-mesothelin, n/a, n/a	DM4	Lysine residues, SPDB disulfide cleavable linker	n/a, 6.5 *mg/kg*, yes	Bayer (57)
BMS-986148	Phase I & II, for treatment of Mesothelin-expressing cancers	anti mesothelin	n/a	n/a	n/a, n/a, n/a	Bristol-Myers (58)
DMOT4039A	Phase I, for treatment of pancreatic and P-OC	hu anti-mesothelin (7D9.v3), n/a, n/a	MMAE	A noncleavable alkyl hydrazide linker	∼ 3.5, 2.4 *mg/kg*, n/a	Genentech, Inc. (59,60)
**Targeting CD22 antigen, a transmembrane sialoglycoprotein functions as an inhibitory receptor for BCR signaling and BCR-induced cell death:**						
Inotuzumab, IO, Ozogamicin, CMC-544	Approved in 2017, for treatment of CD22^+^ ALL	huIgG4 anti CD29(G544), n/a, no	Calich.	Native lysine residues, (AcBut)-N-acyl, Acid-labile hydrazone linker	n/a, 0.05 *mg/kg*, yes	Pfizer (12)
Pinatuzumab vedotin, DCDT2980S, RG7593	Phase II, for treatment of NHL and CLL	huIgG1anti-CD22 (Epratuzumab), n/a, n/a	MMAE	Native cysteines residues, MC-VC-PAB linker	∼ 2.4, 2.4 *mg/kg*, yes	Genentech, Inc. (61)
**Targeting CEACAM5 antigen, labetuzumab, CEA, CD66e, a EGP that has a role in cell adhesion and invasion:**						
IMMU-130, hMN14-SN38, labetuzumab govitecan, labetuzumab-SN-38	Phase II, for treatment of mCRC	huIgG1 anti-CEACAM5 (hMN14), 1.5 *nM*, ADCC	SN-38	Native cysteine residues, CL2A pH sensitive (Benzylcarbonate site) carbonate linker	7-8, 6–10 *mg/kg*, yes	Immunomedics (63–65)
SAR40870	Phase I & II, for treatment of B-Cell Malignancies	huIgG1 anti-CEACAM5, n/a, n/a	DM4	Lysine residues, SPDB disulfide cleavable linker	n/a, n/a, yes	Sanofi (66)
**Targeting Trop-2 (M1S1, TACSTD2 or GA733-1) antigen, a EGP transduces calcium signal has a role in ERK1/2 MAPK pathway which mediates cancer cell proliferation, migration, invasion, and survival:**						
IMMU-132, hrS7-SN-38, Sacituzumab govitecan	Phase III, for treatment of pancreatic cancers, SCLC and TNBC	huIgG1 anti-trop-2 (RS7 or Sacituzumab), 0.564 *nM*, ADCC	SN-38	Native cysteine residues, CL2A pH sensitive carbonate link	∼7.6, 8–10 *mg/kg*, yes	Immunomedics (67–72)
PF-06664178, Trop-2 ADC, RN927C	Phase I, for treatment of OC, NSCLC and breast cancer	Engineered huIgG1anti-Trop-2, 14 *nM*, n/a	PF063801 01	Site-specific transglutaminase tag, AcLys-VC-PABC linker	2.0, n/a, n/a	Pfizer (73)
**Targeting PSMA antigen, a TP2 has known enzymatic activities and acts as a glutamate-preferring carboxypeptidase:**						
PSMA ADC	Phase I & II, for treatment of prostate cancer	hu anti-PSMA, 35.6–46.5 *nM*, n/a	MMAE	Native cysteine residues, VC protease-cleavable linker	n/a, 2.5 *mg/kg*, yes	Progenics (74,75)
MLN2704	Phase I & II, for treatment of prostate cancer	hu anti-PSMA (huJ591), n/a, n/a	DM1	Lysine residues, SPP disulfide cleavable linker	n/a, 60 *mg/kg*, yes	Millennium (76)
**Targeting CD37 (Tetraspanin-26) antigen, a TP3 present on mature B cells, implicates as a signaling death receptor to regulate B/T-cell interactions/proliferation:**						
IMGN529, Naratuximab emtansine	Phase I or II, for treatment of BCL, CLL, NHL	huIgG1anti-CD37 (K7153A), n/a, ADCC and CDC,	DM1	Native lysine residues, SMCC nonreducible thioether linkage	n/a,1.0 *mg/kg*, no	ImmunoGen (78,79)
AGS67E	Phase I, trial for treatment of NHL, DLBCL with high level of CD37 expression	huIgG2κ anti-CD37 (AGS67C or vCD37-9a73), n/a, n/a	MMAE	Native cysteines residues, VC protease-cleavable linker	n/a,1.2 *mg/kg*, yes	Agensys (80–81)
**Targeting CD30 (TNFRSF8) antigen, a tumor necrosis factor:**						
Adcetris, brentuximab vedotin, SGN-35	Approved in 2011, for treatment of HL and ALL.	Chimeric IgG1anti-CD30 (cAC10 or SGN30), n/a,	MMAE	Native interchain cysteine, MC-VC-PABC linker	∼ 4, 1.8 mg/kg, yes	Seattle Genetics (6,7)
**Targeting HER3 antigen, a member of EGFR family RTK, frequently overexpressed in solid tumors, including breast, lung, and colorectal tumors of epithelial origin; it has no active kinase domain itself but is activated through heterodimerization with other members of the EGFR family:**						
U3-1402	Phase I & II, for treatment of HER3-positive metastatic breast cancer	huIgG1anti-HER3(Patritumab)	DXd	n/a	∼8, n/a, n/a	Daiichi Sankyo, Inc. (82)
**Targeting DLL3 antigen, scr-like kinase (Fyn3) acts as a notch ligand for cell-cell communication:**						
Rovalpituzumab tesirine, Rova-T, SC16LD6.5	Phase I & II, for treatment of SCLC	huIgG1 anti-DLL3 antibody (SC-16), 2.6 *nM*, n/a	PBD	Native interchain cysteine, PEG8-va linker, cathepsin-B cleavable dipeptide linker	∼ 2, 0.2 *mg/kg*, yes	Stemcentrx (83)
**Targeting GPNMB antigen, an EGP is involved in differentiation of osteoblasts, and cellular adhesion:**						
Glembatumumab Vedotin (GV), CDX-011, CR011-vcMMAE	Phase II, for treatment of GPNMB-positive breast and melanoma cancer	huIgG2 (CR011), n/a, no	MMAE	Cysteine residues, VC protease-cleavable linker	∼ 4.5, 1.9 *mg/kg*, yes	Celldex Therapeutics (84–87)
**Targeting CD79b antigen, a TP1 on B cells mediates signal transduction cascade activated by BCR:**						
Polatuzumab vedotin, RG7596, DCDS4501A	Phase II, for treatment of NHLs and CLLs	anti-CD79b, n/a, n/a	MMAE	Native cysteine residues, VC protease-cleavable linker	n/a, 2.4 *mg/kg*, yes	Genentech, Inc. (88)
**Targeting GCC antigen, a part of calcium negative feedback system and has a role in cGMP synthesizes from GTP:**						
Indusatumab vedotin, MLN0264, TAK-264, 5F9-vcMMAE	Phase II, for treatment of GI malignancies	IgG1 anti-GCC (TAK-264), n/a, n/a	MMAE	Native cysteine residues, VC protease-cleavable linker	n/a, ∼1.8 *mg/kg*, yes	Millennium (89,90)
**Targeting NaPi2b antigen, a sodium phosphate transporter:**						
Lifastuzumab vedotin, RG7599, DNIB0600A	Phase II, for treatment of NSCLC and ovarian cancer	huIgG1 anti-NaPi2b, 10.19 *nM*, n/a	MMAE	Native cysteine residues, VC protease-cleavable linker	n/a, 2.4 *mg/kg*, yes	Genentech, Inc. (91,92)
**Targeting CA6 antigen, a sialoglycotope of MUC-1 is over-expressed in variety of solid tumors, including breast, ovarian, cervical, lung and pancreatic tumors:**						
SAR566658	Phase II, for treatment of OC, breast, cervical, lung cancers	huIgG1 anti-CA6 (huDS6 IgG1), n/a, n/a	DM4	Native lysine residues, SPDB disulfide cleavable linker	6.5 *mg/kg*	Sanofi (93,94)
**Targeting CD74 antigen, a TP2 on B cells involved in the formation and transport of MHC class II protein:**						
Milatuzumab–doxorubicin, IMMU-110, hLL1-DOX	Phase I & II, for treatment of MM	hu anti-CD74	DOX	Native lysine residues, Acid-labile hydrazone linker	n/a, n/a, yes	Immunomedics (95)
**Targeting CD138 antigen, syndecan1, a type I transmembrane heparan sulfate proteoglycan participates in cell proliferation, cell migration and cell-matrix interactions:**						
BT-062, Indatuximab ravtansine	Phase I & II, for treatment of MM	Chimeric anti-CD138 (nBT062), n/a, n/a	DM4	Native lysine residues, SPDB disulfide cleavable linker	n/a, 2.7 *mg/kg*, yes	Biotest (96)
**Targeting BCMA antigen, a receptor for a proliferation-inducing ligand and B-cell activating factor:**						
GSK2857916	Phase I, for treatment of MM	Engineered afucosylated huIgG1 anti-BCMA, 1 *nM*, ADCC	MMAF	Native cysteine residues, MC noncleavable linker	n/a, n/a, no	GlaxoSmithKine (97)
**Targeting specific myeloma antigen:**						
DFRF4539A, RG7598	Phase I, for treatment of MM	n/a, n/a, n/a	MMAE	n/a	n/a, n/a, n/a	Genentech, Inc. (100)
**Targeting SLAMF7 (CS1) antigen:**						
ABBV-838	Phase I, for treatment of MM	huIgG1 anti-SLAMF7, n/a, n/a	MMAE	Native cysteine residues, VC protease-cleavable linker	n/a, n/a, n/a	Abbvie (101)
**Targeting CD56 antigen, associates with FGFR and stimulates RTKs to induce neurite outgrowth:**						
IMGN901, Lorvotuzumab mertansine, huN901-DM1/BB-10901	Phase I & II, for treatment of CD56+ MM	huIgG1 anti-CD56 (Lorvotuzumab or N901), 0.002 nM, ADCC	DM1	Lysine residues, SPP disulfide cleavable linker	3.7, 2.0 *mg/kg*, n/a	ImmunoGen (102)
**Targeting ENPP3 (CD203c) antigen, a TP2 belongs to a series of ectoenzymes, possess ATPase and ATP pyrophosphatase activities:**						
AGS-16C3F	Phase I & II, for treatment of RRCC	huIgG2k anti-ENPP3 (AGS16-7.8), 0.3–1.1 *nM*, no	MMAF	Native cysteine residues, MC noncleavable linker	∼ 4, 1.8 *mg/kg*, no	Astellas Pharma (103,104)
**Targeting TF (CD142) antigen, a TP and initiator of the coagulation cascade:**						
Humax-TF-ADC, tisotumab vedotin	Phase I & II, for treatment of Multiple solid tumours	IgG1 anti-TF	MMAE	Native cysteine residues, VC protease-cleavable linker	n/a,1.8 *mg/kg*, yes	Genmab (105)
**Targeting TIM1 antigen, a member of the T cell transmembrane IgG and mucin family, which plays critical roles in regulating immune cell activity especially regarding the host response to viral infection:**						
CDX-014	Phase I & II, for treatment of RCC	huIgG1anti-TIM1	MMAE	Native cysteine residues, VC protease-cleavable linker	n/a, n/a, n/a	Celldex Therapeutics (106)
**Targeting FOLR1 antigen, a membrane-bound protein regulates transport of the vitamin B9 into cells:**						
IMGN853, mirvetuximab soravtansine	Phase I, for treatment of folate receptor alpha (FRα)-positive cancer, *e.g*., relapsed EOC	FRa-binding antibody	DM4	Native lysine residues, Sulfo-SPDB disulfide cleavable linker	n/a, 6 *mg/kg*, yes	ImmunoGen (17,107–110)
**Targeting MUC16 (CA-125) antigen, a member of the mucin family GP that acts as a lubricating barrier against foreign particles and infectious agents on the apical membrane of epithelial cells:**						
RG7458, Sofituzumab Vedotin, DMUC5754A	Phase I, for treatment of ovarian and pancreatic cancer	IgG1anti-MUC16 (OC125), n/a, n/a	MMAE and MMAF	Native cysteine residues, MC-VC-PABC linker	n/a, 2.4 *mg/kg*, yes	Genentech, Inc. (111)
**Targeting CanAg antigen, is a novel glycoform of mucin family GP:**						
IMGN242, HuC242-DM4, cantuzumab ravtansine	Phase I, for treatment of Non-colorectal and Pancreatic Cancer	hu anti-CanAg (C242 or cantuzumab), n/a, n/a	DM4	Native lysine residues, SPDB disulfide cleavable linker	n/a, n/a, yes	ImmunoGen (112)
**Targeting Ckit (CD117 or SCFR) antigen, a TP and RTKs having a key role in the regulation of cell differentiation and proliferation:**						
LOP628, Anti c-KIT ADC	Phase I, for treatment of AML and solid tumors	huIgG1anti-(c-Kit), n/a, n/a	DM1	Native lysine residues, SMCC noncleavable thioether linker	n/a, n/a, no	Novartis (113)
**Targeting EphA2 antigen, belonging to ephrin receptor subfamily of the RTKs family regulating cell migration, adhesion, proliferation and differentiation:**						
MEDI-547, MI-CP177	Phase I, for treatment of relapsed or refractory solid tumors associated with EphA2 expression	huIgG1 anti-EphA2 (1C1), 1nM, n/a	MMAF	Native cysteines residues, MC noncleavable linker	4, 6.0 *mg/kg*, no	Medimmune (114,115)
**Targeting Nectin 4 (PVRL4) antigen, a TP1 and member of a family of cellular adhesion molecules, involved in Ca2+-independent cellular adhesion:**						
ASG-22ME, AGS-22M6E, anti-nectin-4 ADC, Enfortumab vedotin	Phase I, for treatment of MUC	huIgG1 anti-nectin-4 (AGS-22M6) 0.01 *nM*, n/a	MMAE	Native cysteines residues, VC protease-cleavable linker	n/a,1–3 *mg/kg*, yes	Astellas Pharma (116,117)
**Targeting SLTRK6 antigen, belonging to the integral TPs(SLITRK) with LRR:**						
AGS15E, anti-SLITRK6 ADC	Phase I, for treatment of MUC	huIgG2γ anti-SLITRK6, n/a, n/a	MMAE	Native cysteines residues, VC protease-cleavable linker	n/a, n/a, yes	Agensys (119)
**Targeting HGFR (cMet) antigen, RTKs for hepatocyte growth factor:**						
ABBV-399, Telisotuzumab vedotin	Phase I, for treatment of c-Met-expressing NSCLC	Engineered huIgG1 without the agonist activity associated with c-Met (ABT-700), 0.2 to 1.5 *nM*, ADCC and c-Met inhibition & downstream signaling molecules	MMAE	Native cysteines residues, VC protease-cleavable linker	∼3.1, 3 *mg/kg*, n/a	Abbvie (120–123)
**Targeting FGFR2 antigen, type 2 RTKs with a role in both embryonic development and tissue repair:**						
BAY1187982, anti-FGFR2 ADC, Aprutumab ixadotin	Phase I, for treatment of FGFR2-positive human malignancies	huIgG1anti-FGFR2 isoforms FGFR2-IIIb and FGFR2-IIIc (BAY 1179470), 75 *nM*, n/a	MMAE	Lysine side chains and a noncleavable linker	∼4, n/a, yes	Bayer (124)
**Targeting C4.4a (LYPD3) and uPAR antigen, glycosylphosphatidylinositol (GPI)-anchored proteins:**						
BAY1129980, Lupartumab amadotin, anti-C4.4a ADC	Phase I, for treatment of LSCC	huIgG1anti-C4.4A, 60 *nM*, n/a	MMAE	Native cysteine residues, noncleavable alkyl hydrazide linker	∼4, 1.9 *mg/kg*, n/a	Bayer (125)
**Targeting p-Cadherin (Cadherin 3) antigen, a cell-surface protein and member of the cadherin family plays a role in cell adhesion, motility, invasion, and proliferation:**						
PCA062	Phase I, for treatment of TNBC; head and neck & esophageal cancers	IgG1 anti-P-cadherin, n/a, n/a	DM1	Native lysine residues, SMCC noncleavable thioether linker	n/a, n/a, n/a	Novartis (126)
**Targeting 5T4 (TPBG) antigen, a EGP correlated with increased invasiveness:**						
PF-06263507, anti-5T4 ADC	Phase I, for treatment of lung and breast cancer with 5T4 expression	huIgG1 anti-5T4	MMAF	Native cysteine residues, MC noncleavable linker	n/a,4.34 *mg/kg*, no	Pfizer (127)
**Targeting STEAP1 antigen, cell-surface protein is predominantly expressed in prostate tissue:**						
RG7450, DSTP3086S, Vandortuzumab vedotin, STEAP1 ADC	Phase I, for treatment of mCRPC	huIgG1 anti-TEAP1(MSTP2109A), 2.4 *nM*, n/a	MMAE	Native cysteine residues, MC-vc-PAB linker	1.8–2.0, 2.4 *mg/kg*, yes	Genentech, Inc. (128–131)
**Targeting PTK7 antigen, RTKs 7 presents on TICs in the Wnt signaling pathway:**						
PF-06647020, h6M24-vc0101, PTK7-targeted ADC	Phase I, for treatment of NSCLC, TNBC and OC	huIgG1anti-PTK7 (h6M24) 0.002 *nM*, n/a	Aur0101	Transglutaminase tag (LLQGA) located at the C-terminus of the antibody heavy chain, cleavable VC-PABC-linker	4, 1.5 *mg/kg*, yes	Pfizer (132,133)
**Targeting Ephrin-A4 (EFNA4) antigen, RTKs modulate signaling pathways that impact cell fate decisions during embryogenesis and adult tissue homeostasis:**						
PF-06647263	Phase I, for treatment of TNBC and OC	huIgG1anti-Ephrin-A4 (E32), n/a, n/a	Calich.	Native lysine residues, Hydrazone–CM1(Hydrazone acetyl butyrate)	4.6, 0.08 *mg/kg*, yes	Pfizer (113,134)
**Targeting LIV1(SLC39A6 or ZIP6) antigen, a member of the zinc transporter family playing a key role in tumor cell progression and metastasis:**						
SGN-LIV1A, anti-LIV-1	Phase I, for treatment of metastatic breast,	huIgG1 anti-LIV1(hLIV22), 4.6 *nM*, n/a	MMAE	Native cysteine residues, VC protease-cleavable linker	4, n/a, yes	Seattle Genetics (135)
**Targeting TENB2 antigen, a prostate cancer target associated with the progression of poorly differentiated and androgen-independent tumor types:**						
Anti-TENB2 ADC	Phase I, for treatment of prostate cancer	ThioMab version of the anti-TENB2 antibody (Pr1), 2.3 *nM*, n/a	MMAE	Native lysine residues, protease-labile VC-PABC-linker	2, n/a, n/a	Seattle Genetics (131,139)
**Targeting ETBR antigen, a G-protein coupled receptor that can activate RAF/MEK signaling:**						
RG7636, DEDN-6526A	Phase I, for treatment of melanoma	huIgG1 anti-ETBR, n/a, n/a	MMAE	n/a	n/a, 2.4 *mg/kg*, n/a	Genentech, Inc. (140)
**Targeting integrin v3 antigen:**						
IMGN-388	Phase I, for treatment of NSCLC and prostate cancer	huIgG1anti-Integrin v3	DM4	Native lysine residues, SPDB disulfide cleavable linker	n/a, 3.5 *mg/kg*, n/a	ImmunoGen (141)
**Targeting crypto antigen, belonging to the EGF-CFC family of growth factor-like molecules, playing a key role in signaling pathways of certain transforming growth factor-beta super-family members:**						
BIIB-015	Phase I, for treatment of breast, ovary, stomach, lung, and pancreas Cripto-expressing tumor cells	huIgG1 anti-Cripto (BIIB015), n/a, n/a	DM4	Native lysine residues, SPDB disulfide cleavable linker	n/a, n/a, n/a	Biogen (142)
**Targeting AGS-5 (SLC44A4) antigen, a sodium-dependent transmembrane transport protein:**						
ASG-5ME	Phase I, for treatment of pancreatic, prostate and gastric cancers	huIgG2 anti-AGS-5, n/a, n/a	MMAE	Native cysteine residues, VC protease-cleavable linker	n/a, n/a, n/a	Seattle Genetics/Astellas (143)
**Targeting LY6E antigen, an interferon (IFN)-inducible glycosylphosphatidyl inositol (GPI)-linked cell membrane protein:**						
RG7841, DLYE5953A	Phase I, for treatment of HER2–breast cancer and NSCLC	n/a, n/a, n/a	MMAE	Native cysteine residues, VC protease-cleavable linker	n/a, n/a, n/a	Genentech, Inc. (144)
**Targeting AXL (UFO) antigen, a member of the TAM (TYRO3, AXL and MER) family of RTK, playing a key role in tumor cell proliferation, survival, invasion and metastasis:**						
HuMax-Axl-ADC	Phase I, for treatment of multiple solid tumors	huIgG1anti-AXL, n/a, n/a	MMAE	Native cysteine residues, VC protease-cleavable linker	n/a, n/a, n/a	Genmab (145)
**Targeting CD205 antigen, a type I C-type lectin receptor normally expressed on various APC and some leukocyte sub-populations:**						
MEN1309/OBT076	Phase I, for treatment of NHL	huIgG1 anti-CD205, n/a, n/a	DM4	Native lysine residues, SPDB disulfide cleavable linker	n/a, n/a, yes	Menarini Ricerche (146)
**Targeting CD25 (IL-2R alpha) antigen, a TP and tumor-associated antigen (TAA), expressed on certain cancer cells:**						
ADCT-301, anti-CD25-PBD ADC	Phase I, for treatment of AML, ALL, relapsed HL and NHL with CD25-positive	huIgG1against CD25, n/a, n/a	PBD	Cleavable linker	n/a, n/a, n/a	ADC Therapeutics S.A. (147)
**Targeting LAMP-1 antigen, playing a key role in cell-cell adhesion and migration:**						
SAR428926	Phase I, for treatment of HER2 negative breast expansion in LAMP-1 positive TNBC	huIgG1anti-LAMP1(Ab-1)	DM4	Lysine residues, SPDB	n/a, n/a, n/a	Sanofi (148)
**Targeting MN/CA IX antigen, a TGP expressed in some human carcinomas and appears to be involved in cancer cell proliferation and transformation:**						
ADC BAY79-4620, MN-IC	n/a	huIgG1 anti-MN/CA IX, n/a, ADCC	MMAE	Native cysteine residues, VC protease-cleavable linker	n/a, n/a, n/a	Bayer (149)

Not available (n/a), Relapsed B-cell non-Hodgkin's lymphoma (B-NHL), Acute myeloid leukemia (AML), Mertansine (DM1), Calicheamicin (calich.), N-succinimidyl 4-(N-maleimidomethyl) cyclohexane-1carboxylate (SMCC), Hydrazone acetyl butyrate (AcBut), Uterine Serous Carcinoma (USC), Tumor-Associated Antigen (TAA), Valine-citrulline-seco (vcseco), Renal Cell Carcinoma (RCC), clear cell Renal Cell Carcinoma (ccRCC), Mantle-Cell Lymphoma Diffuse (MCLD), Non Small-Cell Lung Cancer (NSCLC), Receptor tyrosine kinases (RTKs), Recurrent Glioblastoma Multiforme (GBM), Transmembrane Protein (TP), CD27 ligand (CD27L), Epidermal growth factor receptor variant III (EGFRvIII), Glioblastoma multiforme (GBM), Epithelial Ovarian Cancer (EOC), Head and Neck Squamous Cell Carcinomas (HNSCC), Auristatin F-hydroxypropylamide (AF-HPA), Polyacetal-based polymer (Fleximer®), Non-natural amino acid linker para-acetyl-phenylalanine (pAcF), Amberstatin, a short polyethylene glycol (PEG) spacer terminated by an alkoxyamine (AS269).

B Cell Receptor (BCR), Chronic Lymphocytic Leukemia (CLL), Prostate-specific membrane antigen (PSMA), Maleimido-[short PEG]-Lys-PABOCO-20-O (CL2A), Metastatic colorectal cancer (mCRC), Carcinoembryonic Antigen Related Cell Adhesion Molecule 5 (CEACAM5), Trophoblast cell-surface antigen 2 (Trop-2), Tumor-Associated Calcium Signal Transducer (TACSTD2), Gastric Antigen 733-1 (GA733-1), Malignant Pleural Mesothelioma (MPM), Platinum-resistant ovarian cancer (P-OC).

Target sodium phosphate transporter 2b (NaPi2b), Transmembrane cell surface receptor guanylyl cyclase C (GCC), Delta-like protein 3 (DLL3), polyethylene glycol spacer (PEG8), Selective Catalytic Reduction (scr), Metastatic Urothelial Cancer (MUC), B-Cell Maturation Antigen (BCMA), DX-8951 a derivative of the camptothecin analog exatecan (DXd).

Folate receptor 1(FOLR1), Maleimidocaproyl-valine-citrulline-(MC-VC-PABC), Carbohydrate antigen 125 (CA-125), Mucin 16 (MUC16), A high molecular weight mucin-type glycoprotein (CanAg), Erythropoietin producing hepatoma A2 receptor (EphA2 or EPHA2), Ectonucleotide pyrophosphatase/phosphodiesterase family member 3 (ENPP3), Poliovirus receptor related protein 4 (PVRL4), 2 N-terminal Leucine-Rich Repeat (LRR), Human Tissue Factor (TF), Stem Cell Factor Receptor c-Kit (SCFR).

Hepatocyte Growth Factor Receptor (HGFR), Structural homolog of the urokinase-type Plasminogen Activator Receptor (uPAR), Tumor-associated antigen (C4.4a), Lung Squamous Cell Carcinoma (LSCC), Fibroblast growth factor receptor type 2 (FGFR2), Ovarian Cancers (OC), Trophoblast Glycoprotein (TPBG), metastatic Castration-Resistant Prostate Cancer (mCRPC), - transmembrane epithelial antigen of the prostate-1 (STEAP1), Anti-solute carrier family 39 zinc transporter member 6 (SLC39A6; LIV-1; ZIP6), Anti-Endothelin B Receptor (ETBR), Auristatin-0101 (Aur0101).

Lymphocyte antigen 6 complex locus E (Ly6E), Antigen-Presenting Cell (APC), α subunit of the interleukin-2 receptor (IL-2R alpha), Lysosome-Associated Membrane Protein 1 (LAMP1).

Clinical efficacy of the ADCs arises following accurate selection of four parameters including tumor targeting, antibody, cytotoxic payload, and method of antibody linkage to the payload. The precise selection of each parameter can be achieved through the knowledge gained from the previous studies and established ADCs, and is discussed here.

## Tumor markers in ADCs

**Figure 2.**
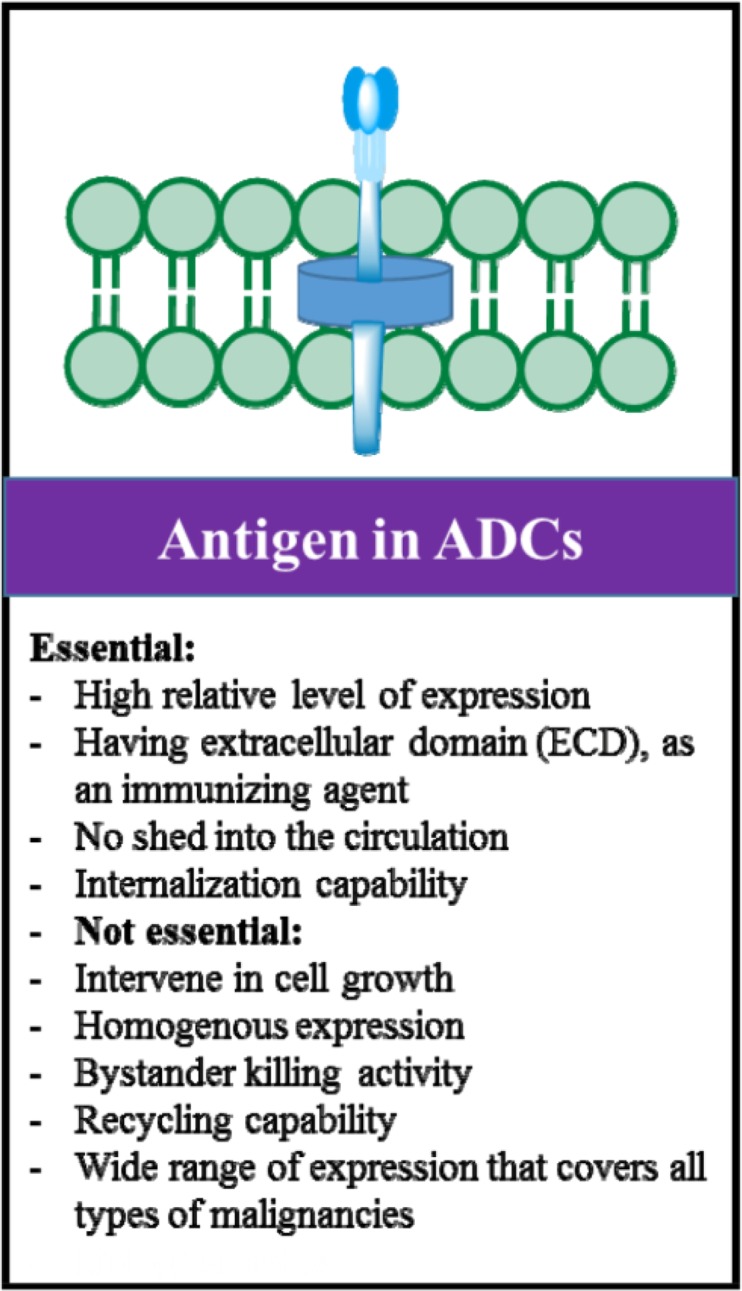
Main considerations in selecting tumor markers for ADC design and development.

The important aspects of tumor markers in ADCs are demonstrated in figure 2. An antigen with expression pattern slightly greater in tumor cells compared to healthy cells is sufficient to induce ADC activity. However, like other targeted drug delivery systems, the number of cell surface tumor markers can be a key determinant of ADC activity 17. The targets for ADC do not necessarily intervene in cell growth. ADCs tumor-suppressive function is mainly mediated through tumor marker potency for ADC internalization compared to the inhibition by blocking the cell growth 1,18–20. However, target biological roles such as those involved in cell division pathway (*e.g*. CD30 and CD70 tumor necrosis factor signaling) can be considered as an advantage for ADC efficacy. Accordingly, the currently employed targets and their biological roles are listed in table 1.

For instance, glembatumumab vedotin is an ADC against an extracellular domain of non-metastatic B melanoma-associated glycoprotein (GPNMB) that is aberrantly expressed in various carcinoma including hepatocellular 21, melanoma 22, gliomas 23, and two specific breast cancer types, Basal-Like Breast Cancer (BLBC) and Triple Negative Breast Cancer (TNBC) 24. The GPNMB do not represent a high relative level of expression in all aforesaid carcinoma. One important property that may make GPNMB a potential therapeutic target for ADCs, originates from its biological role in MAPK/ERK pathway, as GPNMB expression can be upregulated by MAPK/ERK inhibitors 25.

From the structure standpoint, a relevant antigenic determinant on cell surface membranes, termed Extracellular Domain (ECD), is required as an immunizing agent for antibody generation 19. However, the potential of ECD to be shed into the circulation must be considered. The shed ECDs can potentially bind to ADC and consequently reduce the targeted delivery into the tumor cells 19.

A further concern in the selection of the target for ADC is related to the homogeneity or heterogeneity expression of the tumor marker on the tumor cell surface. Homogenous expression of the tumor targets has been demonstrated to be more in favor of ADC targeting than those expressed heterogeneously 26. However, heterogeneous antigen expression can particularly be beneficial for those ADCs that possess bystander killing activity 26–28. Bystander killing activity is referred to the potency of therapeutics delivery system in killing neighboring cells independently of targeted therapy assignment. This effect can be raised through reactive oxygen species or some cytotoxic metabolites that may be excreted from the tumor-targeted cells 26–29. As a result, recycling capability of a tumor marker would enhance bystander killing activity as it may promote leakage of ADC and metabolites to the neighboring cells. However, according to the reports, an extra recycling property is not desirable as in further Bystander activity (Ba), the greater side effects are predicted 30,31.

The promising future of the ADCs supports extensive studies to look for a potent ADC target with a wide range of expression, from earliest cell recognizable lineage to maturation. This represents an exquisitely selective target that covers all types of malignancies. CD19 is a good example of such target that is highly expressed in B-cell and the vast majority of Non-Hodgkin lymphomas (NHLs), and B-cell Acute Lymphoid Leukemia (B-ALL) (99%) 7,32–35. As shown in table 1, CD19 has been marked as a target to produce ADCs, including SAR3419 7,34,35, SGN-CD19A 32, MDX-1206 36, and ADCT-402 33.

## Antibodies in ADCs

**Figure 3.**
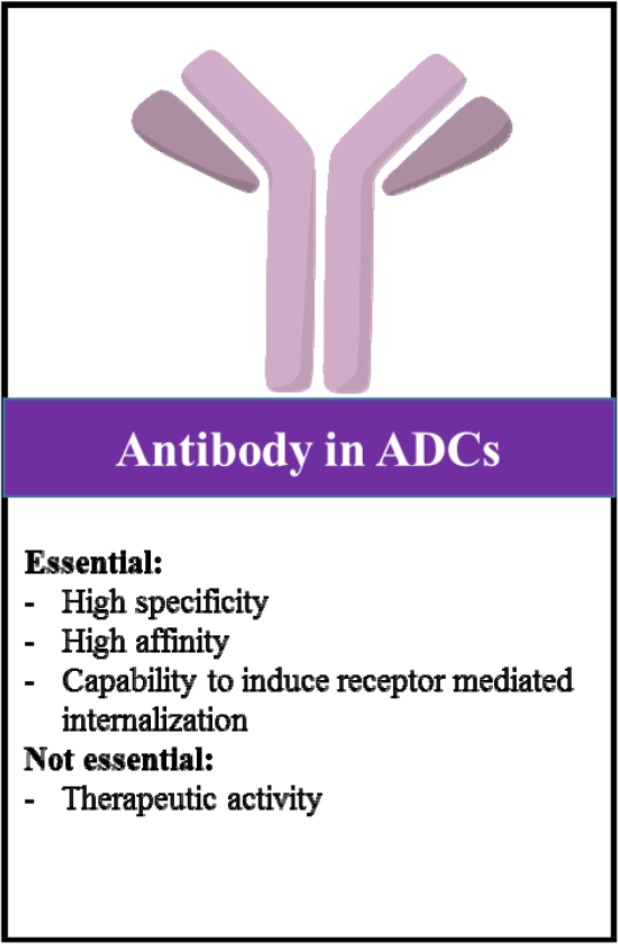
Main considerations in producing antibodies for ADC design and development.

**Figure 4.**
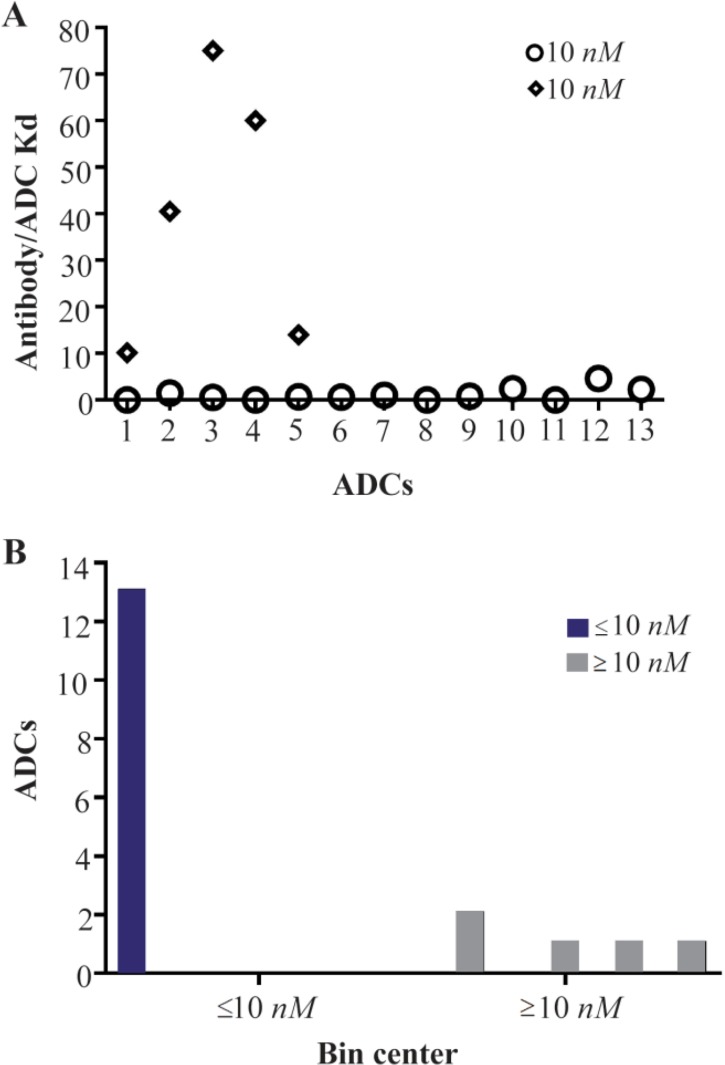
Kd frequency distribution (a) and histogram data (b) of current ADC in clinical development (Table S1, n=13). Antibody affinities (Kd) that have been used in current ADC in clinical development were classified into either ≤10 *nM* or ≥10 *nM* groups. The average Kd and standard deviation of ≤10 *nM* group was 1.12 and 1.3 and for ≥10 *nM* group was 39.9 and 28.2, respectively. Median Kd of ≤10 *nM* group and ≥10 *nM* groups was 0.7 and 40.5, respectively. Average Kd was significantly different between two groups (p<0.05). The frequency distributions of Kd in ≥10 *nM* groups are more than ≤10 *nM* groups (a).

Antibody component in ADCs undertakes both roles including being a carrier and targeting agent. The main aspects of the antibody in ADCs are demonstrated in figure 3. High specificity of targeting and minimal immunogenicity are the main characteristics for Ab component in ADCs. These prevent antibody cross reactions to other antigens, avoiding both toxicity and removal/elimination of the ADC before reaching to the tumor. The high affinity of the Ab for efficient uptake into target cells is another important factor in ADC design 30,54–56. To the best of our knowledge, there is no substantial report about optimal or even minimum required binding affinity (Kd) of antibody component. In figure 4, a binding affinity less than 10 *nM* (Kd<10 *nM*) is commonly needed for the Ab component and accordingly for an effective ADC, based on frequency distribution histogram. The affinity of the antibody to its immunogen can affect the property of antibody which is termed as receptor-mediated antibody internalization. Receptor-mediated antibody internalization is a key mechanism underlying antibody endocytosis that is induced through antibody binding to its specific antigen 77. It has been reported that, alternative antibodies against the same immunogen can exhibit different rates of internalization 19. Rapid internalization can raise both ADC efficacy and safety simultaneously, since it reduces the opportunity of the ADC for off-target release 1,98.

In addition to rapid internalization as a prerequisite for an antibody, the route by which antibody is internalized should be also considered, because it can potentially influence ADC processing 99. For instance, Clathrin-coated Pit-mediated receptor internalization (caveolae pathway), at least in some cases, has been reported to traffic ADC to the cells. In caveolae pathway, ADC is directed to the Golgi or endoplasmic reticulum (Non-proteolytic compartments) instead of endosomes or lysosomes (Proteolytic compartment of the cells) 118. ADC’s traffic to the non-proteolytic compartments may impede its proteolytic process to release effective metabolites 6. Antibody capability to induce receptor mediated internalization is somewhat a mandatory requirement in design of new generation of ADCs. Antibody with low internalization rate has no desired therapeutics index even for the tumors expressing high levels of surface antigen 99. To compensate inefficient internalizing of ADC, a much more potent drug and high stable linkage chemistry (linkage between the antibody and drug moiety) are required that would be discussed in next sections.

Optimal pharmacokinetic (PK) properties including longer half-life is another aspect of the antibody component in ADC design 30,54,55. It has been reported that Ab with longer half-life show high elimination and rapid clearance of the ADC in plasma 136. As shown in table 1, it is not compulsory for a mAb itself to represent therapeutic activity in the ADC. However, therapeutic activity of the mAb is a desirable property besides killing activity mediated by the cytotoxic payload 137,138.

Antibody therapeutic activity is usually mediated *via* immune-mediated effector functions such as Antibody-Dependent Cellular Cytotoxicity (ADCC), Antibody-Dependent Cellular Phagocytosis (ADCP), Complement Dependent Cytotoxicity (CDC), and cytokine signaling modulation in terms of inhibition or induction (Table 1). Such therapeutic activities can be further employed to design ADCs with enhanced cell killing activity 8–11,43,120–123. According to the obtained data in table 1, isotype 1 immunoglobulin (IgG1) seems to be prone to induce immunotherapeutic activity.

In this regard, many attempts have been made to engineer mAbs with therapeutic activity. For instance, the Fc domain affinity of anti-CD19 targeting antibodies for the FcγRIII has been enhanced, either by Fc glycolengineering approaches, *e.g*. MEDI-55 150 and MDX-1342 151 or amino acid substitution, *e.g*. XmAb5574 152 and XmAb 5871 or MOR-208 35,153. Such modification resulted in an increase of ADCC activity in antibody. To the best of our knowledge, the above engineered antibodies have not been used for designing ADCs yet. However, there are some reports of ADCs which have employed a combination/fusion of two engineered antibody fragments. Such fusion antibodies are termed as bispecific Antibody (bsAb), while ADCs designed from the bsAbs were named bispecific ADC (bsADC) 154. Blinatumomab and AFM11 are typical bispecific antibodies, two fusions of anti-CD19 scFv and anti-CD3 scFv, which were engineered to enhance CD19-positive cells killing activity through induction of T or NK cytotoxic immune effector cells 35,155. A derivative of blinatumomab has been also constructed to induce the controlled T cell activation, named ZW38 156. The ZW38 was conjugated to a microtubule cytotoxic agent for the preparation of a novel class of bsADC capable of mediating T cell cytotoxicity 156. Another bsADC, B10v5x225-H-vc-MMAE (Monomethyl auristatin EMMAE), has been recently developed to simultaneously target EGFR and c-MET which are two tyrosine kinases receptors correlated with tumor growth and metastasis 157,158. B10v5x225-H-vc-MMAE contains a bsAb from fusion of anti c-MET Fab fragment and anti-EGFR scFv that was engineered to represent low affinity to EGFR which is a ubiquitous tissue antigen 157. The side effect of B10v5x225-H-vc-MMAE can be avoided to some extent due to attenuated affinity toward EGFR receptors in healthy cells 157. Bridging a rapidly internalizing protein with a tumor specific marker is also another recent method to construct bsAb, *e.g*., anti HER2 crosslink to prolactin cytoplasmic domain receptor 159 with the ability to improve internalization and cell killing activity of the bsADC.

## Cytotoxic payloads in ADCs

**Figure 5.**
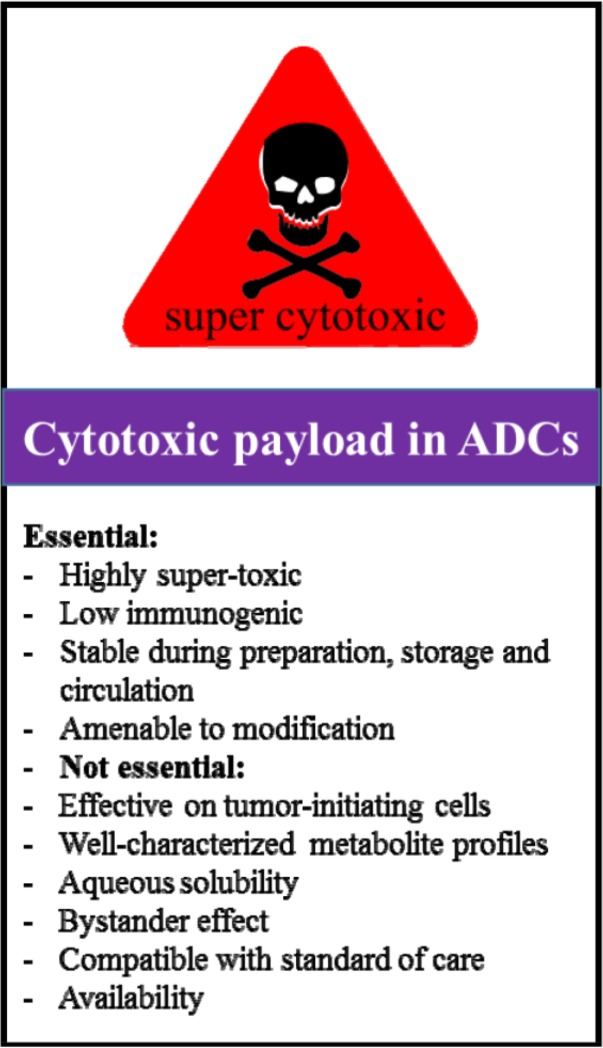
Main considerations in choosing cytotoxic payloads for ADC design and development.

Briefly, cytotoxic payloads for new generation of ADCs should meet many of the criteria as outlined in figure 5. Antibody component in ADCs is incapable of carrying a large number of cytotoxic payload due to its structure. Therefore, the cytotoxic payload in the new generation of ADCs must be highly super-toxic to eradicate majority of the tumor cells even with minimal payload delivery 160. The rate of mAb uptake by tumor cells is approximately less than 0.003–0.08% of injected dose per gram in a tumor 54,55. Furthermore, low expression and poor internalizing activity of the most tumor-associated antigens can cause negligible ADC delivery to the tumor target cells. Hence, ADCs equipped with highly super-cytotoxic payload are imperative, because they must show therapeutic effect while having limited release. According to the reports, a highly cytotoxic agent should exhibit an IC50 of about 10 *nM* or less obtained from an examination with KB cells upon a 24-*hr* exposure time 30,54,55,161. A highly super cytotoxic payload can be originated from plant, animal or microorganisms; in this regard, the most important issue can be the finding of cytotoxic payloads with negligible immunogenic potential in the body. In new generation of ADCs, such cytotoxic payloads are likely to be chemical anti-cancer drugs since experimental evidence confirmed that they are less immunogenic than glycol/peptide cytotoxic agents when circulating in the blood. Some anticancer drugs such as doxorubicin (DOX), mitoxantrone, and etoposide are impaired under hypoxic condition; a condition appeared in solid cancer cell population 162,163. Hence, needless to say, those drugs may not be considered as cytotoxic payloads.

Taking a look at current cytotoxic drugs (Table 1) shows that they generally affect DNA synthesis or cell division to block cell proliferation (mitosis) 38,98. Monomethyl auristatin derivatives which bind to tubulin and are able to inhibit microtubule assembly/polymerization (IC50=10–500 *pM*) 32 are the most commonly used cytotoxic drugs in ADC design with approximately 50% share of the field (Table 1). Maytansinoids derivatives (∼30%), pyrrolobenzodiaze-pine (∼7%), camptothecin analogs (∼6%), n-acetyl-γ-calicheamicin (4%), duocarmycin (DUO) (∼3%) and doxorubicin (∼1%) are the other abundant cytotoxic payloads (Table 1). The above cytotoxic compounds are 100 to10000 folds more potent *in vitro* than typical chemotherapeutic agents and are chosen based on their different actions on cancer and noncancerous cells. DNA modulators have significant effects on malignant cells as they are divided more rapidly than normal cells 163.

Furthermore, a cytotoxic agent of the ADC is better to be studied in an *in vitro* condition to determine whether it is a substrate, inhibitor or inducer of metabolizing enzymes (*e.g*., cytochrome P-450 isozymes (CYPs), and some transporter enzymes like P-glycoprotein) 98. Such studies help to elucidate the *in vivo* factors that may be contributed to the elimination/enhancement of the cytotoxic agent 27,98,164. New studies to introduce new payloads focused on agents against Tumor-Initiating Cells (TICs) 27,164. Such payloads assist to widen the target area and to circumvent potential resistance of cancer cells. Pyrrolobenzodiazepines (PBDs), derivatives of naturally occurring tricyclic antibiotics, duocarmycins, anthracyclines, α-amanitin (a bicyclic octapeptide from the fungus Amanita), and topoisomerase inhibitors including SN-38 are categorized as TIC payloads 1,164.

Rovalpituzumab tesirine is one example of ADC with PBD as a payload (Table 1), that has been reported to have a potency to eliminate pulmonary neuroendocrine TICs at subpicomolar level *in vivo* 83.

The cytotoxic payload should be also stable during preparation or storage and circulation in the blood. Cytotoxic payloads that are not fully stable can potentially be converted to undesirable drug forms during conjugation or storage. Solubility of the cytotoxic agent in aqueous solution is another important criterion in ADC design. Antibody is considered a protein and its conjugation to the cytotoxic agent must be performed in aqueous solutions with minimal organic cosolvents 163,165. Extreme hydrophobicity of payload can potentially change antibodies biological properties, resulting in hydrophobic aggregation of the antibody either during conjugation process or storage 163. The hydrophilicity of cytotoxic payloads will affect cell membrane permeability of parent ADC or its metabolites which may also be beneficial in term of bystander activity 17,26,163,166. However, the ability of cytotoxic payloads to form hydrophobic metabolite after intercellular cleavage of ADC is preferable since the metabolites with more hydrophobic group show better blood clearance and safety 165. According to the reports, about 95–99% of ADC molecules are metabolized before binding to tumor cells 160. This may raise safety concern as it can enhance the potential cytotoxic side effects of ADC. Thereby, the use of cytotoxic payloads with well-characterized metabolite profiles can be an advantage to enhance ADC safety in particular 1,2,167–169.

Cytotoxic payload should present a dominant functional group suitable for linkage to the antibody component of ADC 34. If a dominant functional group does not exist on the cytotoxic agent, at least, it should be amenable to modification, in which a desired substituent is introduced on appropriate sites 170.

The copy number and heterogeneity of antigen expression are the other important issues that must be considered in the selection of cytotoxic agent 30,31. More expression of target antigen may be a reason to apply a cytotoxic agent with low potency. Typically, payloads that promote the bystander effect in cancer cells are more desirable to design ADCs directed for the antigens expressed heterogeneously 26.

The ability to choose specified cytotoxic payloads with mechanism of action compatible with standard of care has been reported to facilitate clinical success of the ADCs in biopharmaceutical market. For instance, microtubule disrupting payloads are commonly chemotherapeutic drugs that are used for the treatment of cancers, including breast, ovarian and prostate cancer 54,55 (Table 1). Both availability in the market and reasonable cost can be alternative rationale for choosing a cytotoxic payload in ADC design 1.

## Linking cytotoxic payloads to antibodies in ADCs

**Figure 6.**
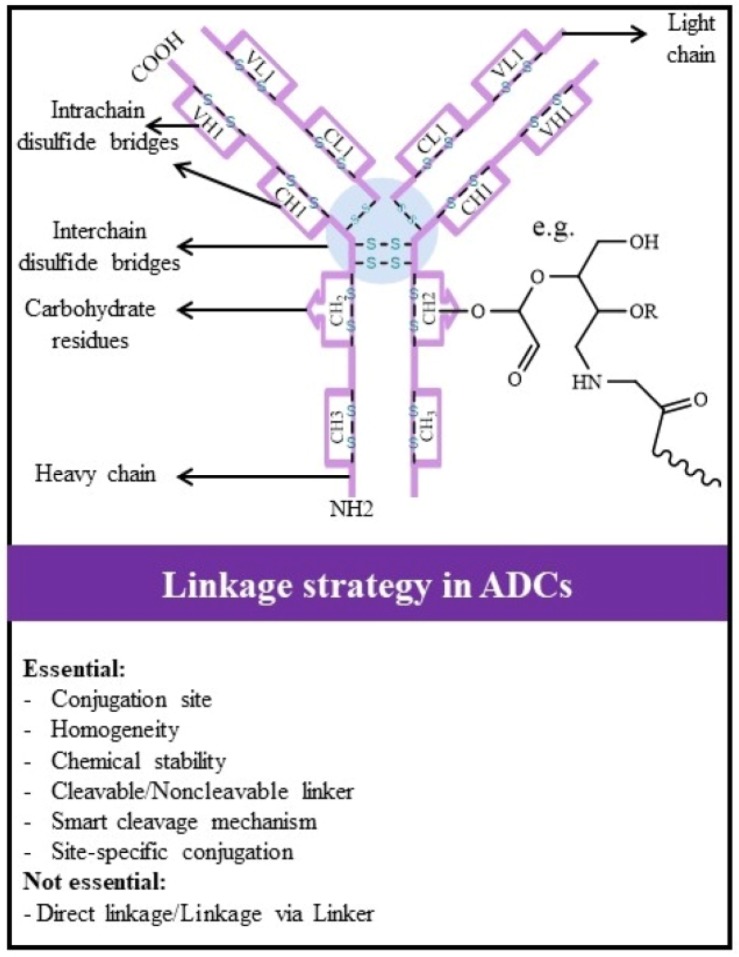
Main considerations for linking cytotoxic payload to antibodies in ADC design and development.

One of the dynamic research fields in ADC design is the study of the methods that are correlated with antibody conjugation to cytotoxic payloads, as it has a great role on balancing between ADC therapeutic efficacy and toxicity 30,31,54. The key concerns in linkage chemistry are demonstrated in figure 6. Conjugation site on antibody component, a well-defined Drug to Antibody Ratio (DAR), homogeneity and linkage stability are the important parameters that need to be considered in conjugation.

In general, interchain disulfide bridges and surface-exposed lysines are the most currently used residues on the antibody for conjugation to cytotoxic payloads, respectively (>50 *vs*. >30%) (Table 1). Hydroxyl groups on carbohydrate structures are the other residues in antibodies that have been rarely used as conjugation sites for ADC (The schematic linkage in figure 6 is an example of this strategy) 1,171.

Theoretically, the linkage of cytotoxic payloads to the surface-exposed lysine of mAb occurs after reduction of ∼40 lysine residues on both heavy and light chain of mAb 172 and it results in 0–8 cytotoxic payload linkages per antibody and heterogeneity with about one million different ADC species 30,173. Cysteine conjugation occurs after reduction of four interchain disulfide bonds and results in eight exposed sulfhydryl groups. Linking drugs per antibody can differ from zero to 8 molecules, generating a heterogeneous population of ADC (Greater than one hundred different ADC species) 30.

Due to low stability and safety properties of the pharmaceutical products with heterogeneous contents, they are complex to be accurately predicted in terms of efficacy or therapeutic window 27,30. Therefore, improvement of conjugation methods to achieve homogeneous ADC is very crucial.

In this case, it is possible to reduce just two of four interchain mAb’s disulfide bonds of cysteine residues through carefully mild reduction conditions, as interchain disulfide bridges are more prone to reduction than intrachain disulfide bridges 171,174,175. However, such mild reduction is not easily possible in practice and a diverse number of cysteines may be reduced (0–4), resulting in a heterogeneous mixture of ADC 30,173. Hence, the production of homogeneous ADCs through payload conjugation with native residues can be laborious. To overcome this limitation, many site-specific conjugation approaches have been developed, in which a known number of cytotoxic payloads are constantly conjugated to defined sites on mAbs. Some of the approaches are explained below:

1. A conjugation through engineered cysteine residues that neither damages antibody fab region nor interferes with Fc-mediated effector functions, called THIOMAB technology 173,176. In THIOMAB technology, the heavy chain alanine 114 is substituted with two or more reactive cysteine residues at a predefined site for conjugation with cytotoxic payload 173. Anti-TENB2 ADC is an example that is prepared by THIOMAB technology and is currently in phase I trial (Table 1).
2. Re-engineering of mAb is able to incorporate with unnatural amino acids, *e.g*. selenocysteine 177, acetyl-phenylalanine 178, and para-azidomethyl-l-phenylalanine 42.
3. Site-specific enzyme-mediated conjugation to genetically engineered antibody is as follows:
  Incorporating a thiolated sugar analogue, 6-thiofucose, to the antibody carbohydrate that introduces new chemically active thiol groups using fucosyltransferase VIII 179,
  Providing a ketone reactive group on antibody glycosylation site by glycotransferases 180,
  Introducing an aldehyde reactive group on the antibody using sialyltransferase 181 or formylglycine-generating enzyme 182,
  Genetically introducing specific glutamine tags to antibody whereby payloads with a primary amine group can be linked to the γ-carbonyl amide group of glutamine tags. Such reaction is catalyzed by a microbial transglutaminase which is capable of recognizing glutamines tags from naturally glutamines residues 73,183–185,
  Providing LPXTG tagged antibodies (A penta-peptide as a substrate for transpeptidation reaction) as specific linkage sites for the oligo-glycine-containing payloads, which are mediated by *Staphylococcus aureus* Sortase A enzyme 186,
  Conjugation of phosphopantetheine-linked payloads to the serine residues of the peptide-tagged antibodies via phosphopantetheinyl transferases catalysis 187,
4. Chemoenzymatic site direct conjugation, *e.g*., providing two azide groups at asparagine 297 (Asn-297) residue in antibody constant region (Fc) is linked with cytotoxic payloads using copper-mediated click reaction 188. The azide functional groups are formed in a selective hydrolysis reaction that is mediated by an Endo-beta-N-acetylglucosaminidase (EndoS) chemoenzyme.

ADC as a potential targeted delivery system must be passed through all hurdles, including blood circulation, antigen binding, internalization, payload release, and eventual payload action. An unstable linkage can lead to premature release of the payload, before reaching the site of action 98. Therefore, reasonable chemical stability must be considered in the design of chemical linkage between cytotoxic payload and antibody.

Although a direct linkage between cytotoxic and antibody components has generally shown more stability in circulation 1,98, conjugation reactions are mostly created with linkers in comparison with direct linkage between cytotoxic and antibody component (Table 1). The choice of proper linkers has been discussed in the related publications devoted to the progress of ADCs 30,31,54,189,190. As shown in table 1, about 50% of the ADCs are using Valine-Citrulline peptidyl (VC) linker. N-succinimidyl 4-(2-pyridyldithio) butyrate (SPDB) (18%), acid-labile hydrazine (10%), maleimidomethyl cyclohexane-1-carboxylate (MCC), maleimidocaproyl (MC) (10%), N-succinimidyl 4-(2-pyridyldithio pentanoate (SPP) and carbonate (3%) linkers are other employed linkers.

Limited drug-linker designs for more than 70 current ADC clinical trials (Table 1) are a dilemma regarding linkage chemistry that may restrict simultaneous development of ADCs against both hematological and solid tumors. Generally, the properties of linkers can be altered by the cytotoxic payload release mechanism 191. Cytotoxic payload in ADC technology must be released into the cell to exert its therapeutic activity, thus ADC linkers should be chosen based on their stability to keep ADC intact during circulation and capable of cleaving inside the targeted cell 191,192. Linker stability is defined based on lack/low level of cleaving agents (*e.g*., protease or reductive agents) in the bloodstream compared to the cytoplasm 163.

The current linkers used in ADCs are also broadly classified as cleavable and noncleavable linkers based on where they are cleaved into the cytoplasm. Cleavable linkers are those containing a conditional cleavage sites sensitive to be cleaved immediately after ADC internalization, such as VC, SPDB, SPP, and hydrazine which can be triggered through protease reactions, glutathione reduction, and acidic pH, respectively 163,164. Noncleavable linkers are stable from early to late endosome transition and their cytotoxic partner is just released by degradation of antibody in lysosomes, *e.g*. MCC and MC linkers that link Ab to the payload via thioether linkage 190.

Characteristics of ADC target such as copy number, internalization rate and level of homogeneity should be considered in conjugation method and linker selection. For instance, ADC with disulfide-linkage has been shown to have more cytotoxic activity than the same ADC with thioether linkage when they were directed to the tumor cell lines expressing a low copy number of targeted antigen 17.

Cleavable linkers may increase the possibility of bystander effect 27. Hence, it is logical to use cleavable linkers in designing ADCs directed for the antigen that is heterogeneously expressed in tumors 26.

*In vivo* adverse effects of ADCs are influenced by the use of cleavable or noncleavable linkers. As in the case of tubulin inhibitor payloads, which is linked through cleavable linkers to the antibody component, *e.g*. SPDB-DM4 (Ravtansine-DM4), or VC-MMAE, peripheral neuropathy can be frequently observed, whereas noncleavable linkers often trigger hematological toxicity, possibly due to an increased dose and interactions with Fcγ receptors on hematopoietic cells 164.

The type of linker plays an important role in ADC catabolite products with regard to processing into targeted cells or metabolizing by clearance mechanisms. The type of ADC catabolites may influence some ADC features such as IC50, Maximum Tolerated Dose (MTD) 192,193, and kill Multidrug Resistance (MDR) expressing cells 192,194.

## Conclusion

ADC is considered exciting and promising antibody-based therapeutics to improve cancer therapy. Growth in the number of registered ADCs in clinical trials (Table 1) represents the pharmaceutical industry interest in investment for research and development in the field, as it has been stated by others 14,15.

The design of an ADC might seem to be not very complex, while several issues must be taken into consideration to complete ADC’s potential as a therapeutic agent for cancer. This might be the main reason for the condition that small number of ADCs have reached the market (Table 1). The major issues associated with the development of ADCs seem to be originated from the factors that interfere with ADCs efficacy and off-target cytotoxicity. The precise selection of all four parameters, *i.e*. tumor marker, antibody, cytotoxic payload, and linkage strategy would be required to prepare a successful ADC.

With regard to ADC tumor markers, they do not have to be involved in tumor growth 1,18,20,31. Therefore, ADC can present therapeutic application in a broad range of tumors. However, an ADC tumor marker should meet at least three criteria of considerable expression level in tumor cells *vs.* normal cells, presenting cell surface immunogen, and being capable of performing ADC internalization.

High specificity, adequate affinity, and receptor-mediated internalization are the major aspects of antibody choice. Efforts to optimize antibody component would be a great idea to translate into improved ADCs. In fact, some major ADCs’ weaknesses including, low efficiency 156, low internalization 159, off-target effect due to the target expression in normal tissues 157, and heterogeneity expression of the target in the tumors can be overcome *via* antibody improvement. Antibody engineering technology for production of alternative bsAbs to design more efficient ADCs (bsADCs) has been proven in several preclinical models 156,157,159. The rationale behind this technology is the fact that the aforesaid ADC’s weaknesses can be solved through ADC designs (bsADCs) operating from improved antibody (bsAb) in terms of affinity, specificity, internalization activity, by enhancing the therapeutic activity or decreasing ADC’s side effects.

Another main concern in the development of ADCs is related to the study of finding cytotoxic payloads that are potent enough with confined DAR (Up to 7 drugs per antibody) 195 to exert therapeutic activity. Having reasonable aqueous solubility, non-immunogenic, as well as stability in storage and bloodstream is a common criterion for choosing cytotoxic payloads.

In contrast, the introduction of innovative methods to modify ADCs cytotoxic payloads with versatile functional groups (*e.g*. thiol, amine groups) is the other interesting subject, as it eases the conjugation process. One further challenge of ADCs is associated with the limitation of linkage and conjugation chemistry to link an optimized number of the payloads to the antibody in predefined location homogeneously.

Interdisciplinary and multidisciplinary works and related studies such as recombinant DNA technology, bioconjugation, and chemistry are the hopeful strategies to get the purpose of achievement in site-specific conjugation and homogeneous ADCs 73,173,176–187,196,197.

Based on promising reports from research to synthesize homogeneous ADCs, it is likely that the first ADC products constructed using site-specific conjugation will be made for cancer therapy that may hold the promise about the future use of ADCs.

Taken together, despite challenges in ADC design, the future of ADCs seems to be much promising as more clinical trials and basic researches conducted on existing ADCs would pave the way to tackle issues regarding tumor marker, antibody, cytotoxic payload, and linkage strategy.
